# The epidemiology of khat (catha edulis) chewing and alcohol consumption among pregnant women in Ethiopia: A systematic review and meta-analysis

**DOI:** 10.1371/journal.pgph.0002248

**Published:** 2023-09-15

**Authors:** Biruk Wogayehu, Tsegaye Demissie, Eskinder Wolka, Mekuriaw Alemayehu, Kassa Daka

**Affiliations:** 1 Department of Public Health, Arbaminch College of Health Sciences, Arbaminch Town, Ethiopia; 2 Department of Public Health, School of Public Health, College of Medicine and Health Sciences, Wolaita Sodo University, Sodo Town, Ethiopia; 3 Department of Epidemiology and Biostatistics, Institute of Public Health, College of Medicine and Health Sciences, University of Gondar, Gondar Town, Ethiopia; London School of Hygiene and Tropical Medicine Faculty of Epidemiology and Population Health, UNITED KINGDOM

## Abstract

The use of khat *(Catha edulis)* and alcohol during pregnancy is a serious public health problem that has been associated with a number of harmful outcomes for both the fetus and the mother’s health. There has been no systematic review with meta-analysis to determine risk factors associated with khat and alcohol use among pregnant women in Ethiopia. Therefore, we aimed to determine the pooled prevalence and risk factors of khat and alcohol use during pregnancy in Ethiopia. This review has been registered in PROSPERO with protocol ID: CRD42023395115. Studies identified from PubMed, Google Scholar, the WHO African Index Medicus, the Cochrane Library, African Journal Online, and Science Direct. Articles published from January 1, 2000 to February 10, 2023 were included. We searched for articles that included any combination of the following key terms: “khat”, “qat”, “alcohol”, “ethanol”, “prevalence”, “factors”, “pregnant” and “Ethiopia”. Two reviewers worked independently to screen studies and extract data. A funnel plot and Egger’s regression test were used to test publication bias. A forest plot was used to present the pooled prevalence and odds ratio with a 95% confidence interval (CI) using the random effect model. I^2^ metrics were used to assess heterogeneity. The meta-analysis was carried out with Stata 14.0 software. Nine hundred sixty-two records were retrieved from different sources, and 23 studies were included in the final analysis. The pooled prevalence of khat use and alcohol drinking during pregnancy was 26.6% (95% CI 17.8, 35.5) and 31.65% (95% CI: 21.8, 41.5), respectively. Partner khat use (OR 5.9 [95% CI (2.4, 14.5)]) was associated factor for khat use during pregnancy. Low educational level (OR 2.54 [95% CI (1.8, 3.5)]), pre-pregnancy alcohol use (OR 3.5 [95% CI (2.6, 4.7)]), unplanned pregnancy (OR 2.7 [95% CI (1.8, 4.0)]), history of abortion (OR 2.3 [95% CI (1.4, 3.7)]), poor social support (OR 3.3 [95% CI (2.0, 5.3)]), and mental distress (OR 2.6 [95% CI (2.0, 3.3)]) were associated factors for alcohol drinking during pregnancy. This review indicated that the magnitude of khat and alcohol use during pregnancy in Ethiopia was high. Targeted interventions for groups of pregnant women at high risk of khat and alcohol use are urgently needed. Community-based health education interventions and point-of-sale warnings are essential to reduce the burden. Future studies should consider the influence of community-level factors on khat and alcohol use during pregnancy.

## 1. Introduction

Prenatal substance use is a major public health issue that has been connected to a number of negative maternal and fetal outcomes [1]. In Ethiopia, the most frequently used substance in pregnancy is alcohol, followed by khat and other illicit substances [2]. Khat (Catha edulis Forsk) is a psychostimulant herbal plant that grows abundantly in East Africa and the Southern Arabian Peninsula, and certain Ethiopian populations have chewed khat leaves for centuries [3]. Khat contains the active chemicals cathinone and cathine, which resemble amphetamine structurally and functionally and share many pharmacologic properties [4]. Khat chewing during pregnancy is associated with the risk of low birth weight (LBW) [5–7], reduced length of limb long bones [8], intrauterine growth retardation [9], and prelabor rupture of membranes [10].

Alcohol use during pregnancy can result in a variety of health problems for both the child and the mother. Alcohol is a teratogen that can easily cross the placenta, causing permanent damage to the developing embryo’s and fetus’s brain and other organs. Women who drink alcohol during pregnancy put their child at risk of developing fetal alcohol spectrum disorder (FASD) [11], as well as a variety of other negative pregnancy outcomes such as stillbirth [12], low birth weight [13], premature birth [14], spontaneous abortion [15], and intrauterine growth retardation [13].

A global systematic review and meta-analysis of 328 studies in 50 countries showed that the prevalence of alcohol consumption during pregnancy was 9.8% [16]. Another meta-analysis from the WHO African Region revealed that a wide variation in prevalence, ranging from 8.08% in Nigeria to 32.5% in Zambia [17]. Additionally, a systematic review and meta-analysis study of alcohol use among pregnant women in Sub-Saharan Africa by Addila et al. 2020 [18] reported that the pooled prevalence was 20.8%. Several alcohol use epidemiological studies among pregnant women are available in Ethiopia. However, the results of these studies showed a wide variation of prevalence ranging from 4.10 to 76.70% [19–34] over time and across geographical areas. Similarly, a national study conducted using Ethiopian Demographic Health Survey (EDHS) 2016 data showed that the spatial distribution of alcohol consumption was highly variable across the country. Northwest Ethiopia and Central Ethiopia are the most likely clusters with high rates of alcohol use [25]. A recent review of alcohol use among pregnant women in Ethiopia concluded that the pooled prevalence of alcohol use among pregnant women was 14.1% [35].

In the past few decades, khat use by women was deemed unacceptable, but recently its consumption among women has rapidly grown and become socially accepted [36]. In a national population survey in Yemen by Marwan et al. involving 7343 married women, 40.7% of respondents reported consuming khat while pregnant [37]. Studies on pregnant women in Ethiopia showed that the prevalence of khat use in pregnant women ranges between 5.8% and 65.8% [6, 7, 10, 19, 20, 22, 23, 38–41]. Regional distribution of khat use in Ethiopia can be described as ranging from 15.5% to 37.2% in Harar region [10, 38, 40], 9.9% to 35.8% in Southern Ethiopia [23, 39], 5.8% to 65.8% in Oromia region [19, 20, 29, 41] and 15.3% in Addis Ababa city [6].

Several primary studies conducted in Ethiopia have highlighted the key risk factors associated with khat use during pregnancy, including being Muslim [41], low level of education [41], mental distress [39], family with mental health history [41], partner khat use [39, 41], and alcohol use [39, 41]. Several factors have been found to increase the likelihood of alcohol use during pregnancy. Alcohol use during pregnancy has been found to be significantly associated with: urban residence [21], low educational level [27, 29, 30, 32, 34], history of pre-pregnancy alcohol use [21, 27, 29], khat chewing [21], history of abortion [21, 24, 30], unplanned pregnancy [21, 27, 29, 30], poor social support [24, 27, 29, 30], mental distress [21, 24, 27, 29], and a lack of knowledge about the harmful fetal effects of alcohol use [27, 30]. Besides this, partner alcohol use is one of the factors that contributes to the likelihood of alcohol use [29].

To our knowledge, this is the first systematic review and meta-analysis of the prevalence of khat use and associated risk factors among pregnant women in Ethiopia. The first systematic review and meta-analysis of the prevalence of alcohol use among pregnant women in Ethiopia was conducted by Duko et al. [35] . However, the previous review did not estimate the pooled odds ratio of risk factors associated with alcohol use. Moreover, the previous review did not report the pooled prevalence based on geographical location (region). A comprehensive literature search is a key component of a well-conducted systematic review. Therefore, in the absence of concrete and inclusive evidence on khat and alcohol use during pregnancy, this systematic review and meta-analysis were conducted to determine the pooled prevalence of and factors associated with khat and alcohol use among pregnant women in Ethiopia. Identifying and assessing the risk and protective factors that contribute to khat and alcohol use is fundamental to determining appropriate interventions and treatment.

## 2. Methods

### 2.1 Reporting and protocol registration

The methodology and reporting of this review have been guided by the Preferred Reporting Items for Systematic Reviews and Meta-Analyses (PRISMA; S1 Table) [42]. The review protocol was prospectively registered in the international prospective register of systematic review, PROSPERO with ID: CRD42023395115. This review protocol is available at https://www.crd.york.ac.uk/prospero/display_record.php?ID=CRD42023395115. For the present review, approval from an ethics review board was not required as it focused on summarizing the findings of studies made public. Moreover, data from previously published articles in which informed consent was obtained by the primary investigators were retrieved and analyzed. However, the methods followed clear steps from the literature search to the synthesis of results.

### 2.2 Search strategy

**Fig 1:**
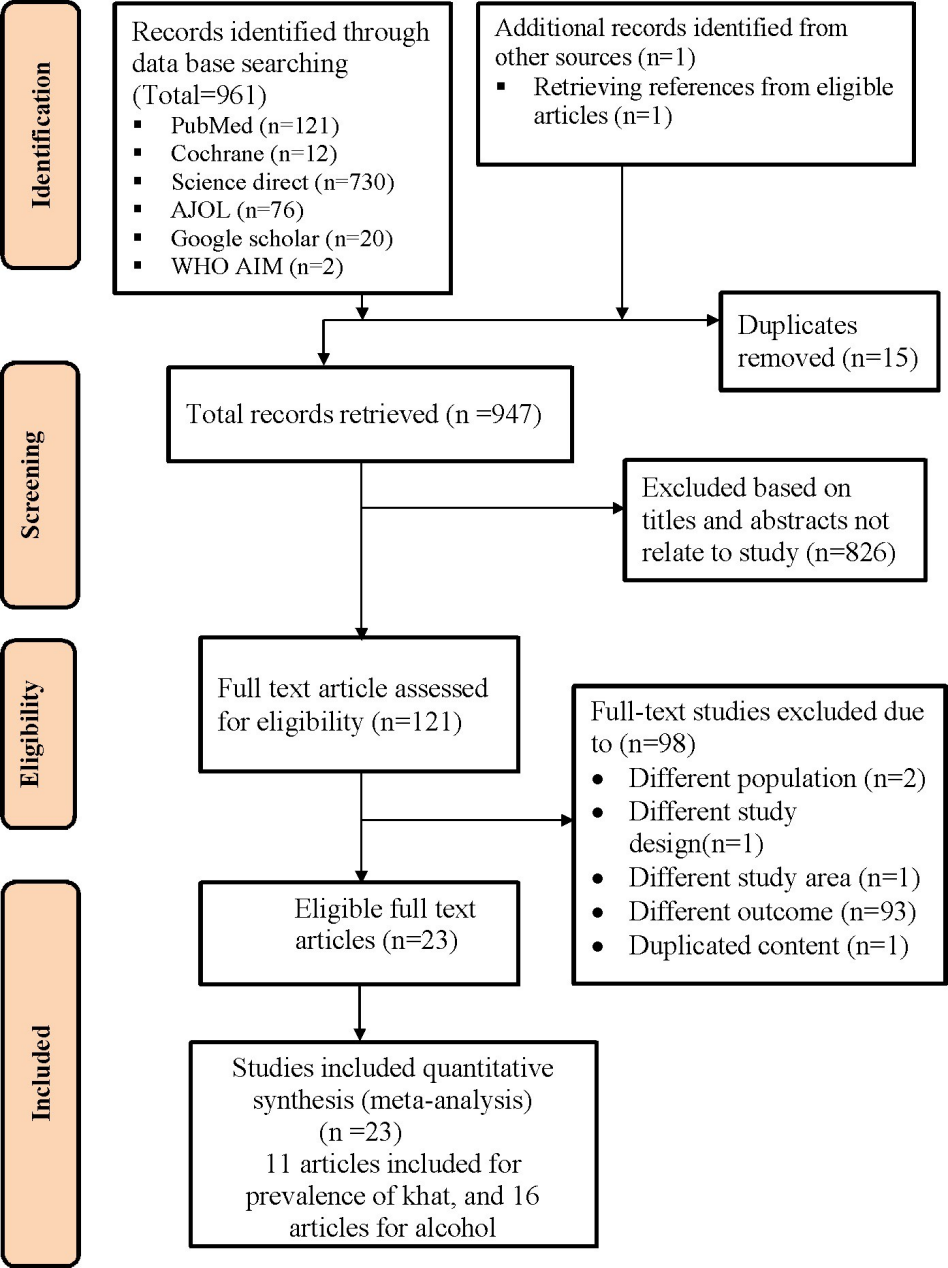
Flow chart showing the search findings for studies on khat and alcohol drinking use during pregnancy in Ethiopia from January 1, 2000 to February 10, 2023.

A comprehensive search was performed in PubMed, Science Direct, Cochrane library, the WHO African Index Medicus (AIM), African journals online (AJOL), and Google Scholar databases to identify relevant studies published on khat chewing and alcohol use among pregnant women in Ethiopia from January 1, 2000 to February 10, 2023. The search for studies was conducted in January 2023. A search strategy based on the combination of relevant terms were conceived and applied. Both key words and medical subject heading (MeSH) terms were used. The following terms and their variants were used for khat: “catha”, “khat”, “qat”, “chat”, “prevalence”, “magnitude”, “factors”, “predictors” .For alcohol, we used the terms “ethanol”, “alcohol”, “binge drinking”, “substance use”, “substance use disorder”, “prevalence”, “magnitude”, “factors”, “predictors”, “pregnant”, “Ethiopia”. A search strategy for each database is provided in S2 Table. To identify further articles, bibliographies from other published reviews and all retrieved papers were cross-checked (Fig 1).

### 2.3 Study eligibility

#### 2.3.1 Inclusion criteria

Studies were included in the review if they fulfilled the following criteria:

- Study design: observational studies (including case-control, cohort and cross-sectional studies).
- Participants: conducted among pregnant women in Ethiopia.
- Outcome measurements: the prevalence of khat or alcohol use and associated factors.
- The main outcome of this review is to estimate the pooled prevalence of khat chewing and alcohol consumption among pregnant women in Ethiopia. In addition, it identified determinants associated with khat and alcohol use during pregnancy. Khat or alcohol use defined as percentage pregnant women who are using any amount of khat or alcohol during pregnancy. Any measurement of khat and alcohol consumption during pregnancy was included.
- Language: published studies written in English.

#### 2.3.2 Exclusion criteria

- Reviews, experimental studies, letters, commentaries and editorials.
- Unpublished studies.
- Duplication studies were also checked and removed before the analysis process started.

### 2.4 Screening

Two researchers (BW and TD) individually chose studies that met the inclusion criteria. Titles and abstracts were reviewed for inclusion, and duplicate citations were eliminated. Following the screening of titles and abstracts according to the inclusion criteria given above, the full texts of possibly eligible papers were collected. To include qualifying research, the entire texts were checked using a standardized and pre-tested procedure. Disagreements were settled by consensus or consultation with a third author (EW). The reasons for non-eligible studies’ exclusion were noted. Articles obtained from electronic databases were imported into Endnote software (version X4) to aid with article selection and citation management.

### 2.5 Data extraction

Data extraction from the included studies was done separately by two reviewers (BW and TD) using a data extraction template developed on Microsoft Excel Spreadsheet 2010. The primary author’s name, study region, study setting, study period, sample size, khat and alcohol prevalence estimates, mean age of participants, response rate, and factors were all collected.

### 2.6 Assessment of methodological quality and risk of bias

Two authors (BW and TD) evaluated the quality of the publications included in the review using the Joanna Briggs Institute (JBI) Critical Appraisal Checklist for Studies Reporting Prevalence Data [43]. Based on nine criteria, the checklist evaluates the methodological quality of prevalence studies. Yes, no, uncertain, or not relevant were possible options.

### 2.7 Data processing and analysis

In this review, the data was extracted using standard Microsoft excel form and then exported into STATA version 14.0 software for analysis. We used to pool outcome results from eligible studies. The pooled prevalence of khat or alcohol use and the pooled odds ratio of associated factors with 95% CI were determined using the random-effects model. The heterogeneity between studies was assessed using the Cochran’s Q chi-squared test statistic and the Higgins and Thompson’s I^2^ statistic. The cut-off I^2^ values of 0%, 25%, 50%, and 75% was respectively represent no, low, moderate, and high level of heterogeneity [44]. Publication bias was assessed through visual inspection of a funnel plot and the result of the Egger test, considering statistically significant at *p*-value less than 0.05. We conducted sub-group analysis and meta-regression analyses to explore the potential sources of heterogeneity. Subgroup analyses were applied based on region (Amhara, Southern Nations, Nationalities, and Peoples’ Region (SNNPR), Oromia, Addis Ababa and Eastern Ethiopia), study setting (community or institution bases study), study year, sample size, data collection tool and the mean age. Univariate meta-regression analyses will be performed with restricted maximum likelihood estimation and includes region (Amhara, SNNPR, Oromia, Addis Ababa and Eastern Ethiopia), study setting (community or institution bases study), study year, sample size, data collection tool and the mean age as covariates. The robustness of the pooled estimates was assessed by sensitivity analysis (using leave-one-out analysis).

## 3. Results

### 3.1 Study selection

Fig 1 shows the steps for searching and retrieving articles. A total of 962 studies were identified through the search of electronic database (PubMed = 121, Cochrane = 12, Science direct = 730, AJOL = 76, Google Scholar = 20, WHO AIM = 2) and other retrieving reference from eligible studies (n = 1). After scanning the reference list of retrieved studies, one new study was discovered. From all identified articles, 15 studies were removed due to duplication while 947 articles were reserved for additional screening. Of these, 826 were removed after reading the title or the abstract since either they were not conducted in Ethiopia or they were not in line with the aim of this review. As a result, 121 full-text studies were accessed and evaluated for eligibility using pre-defined criteria, which resulted in further exclusion of 98 studies due to the study population, study setting, outcome of interest, study design, and duplication. Finally, 23 articles that fulfilled the eligibility criteria were included in the qualitative and quantitative synthesis.

### 3.2 Characteristics of included studies

The main characteristics of all included studies presented in S3A and S3B Table. Overall, 7 studies assessed khat use only [6, 7, 10, 38–41], and 12 studies assessed alcohol consumption only [21, 24–34], and 4 studies assessed both khat and alcohol use during pregnancy [19, 20, 22, 38]. These studies were all cross-sectional and conducted in 5 regions of Ethiopia between 2014 and 2020. Eight studies were conducted in Amhara region [24, 26–28, 31–34], three in Addis Ababa [6, 29, 30], four in Eastern Ethiopia [10, 22, 38, 40], three in SNNPR [21, 23, 39], four in Oromia region [7, 19, 20, 41]. One study was a national survey [25]. Ten studies were conducted in community setting [23, 25, 27, 28, 31–34, 38, 40] and thirteen in health facility setting [6, 7, 10, 19, 20–22, 24, 26, 29, 30, 39, 41]. Ninety-one percent (91%) of studies were published within the last five years (2018–2023).

For khat use, the selected studies included 8698 participants, with a mean sample size of 790 (range 293–1688). For alcohol use, the selected studies included 9820 participants, with a mean sample size of 613 (range 228–1216). Seven of sixteen studies included in alcohol use meta-analysis utilized a standard tool (AUDIT or CAGE) [21, 23, 24, 27, 29–31] for the assessment of alcohol consumption and the remaining nine utilized dichotomous questionnaire [19, 20, 22, 25, 26, 28, 32–34]. For khat use, two factors were extracted from 2 included studies [39, 41]. For alcohol use, ten factors were extracted from 8 included studies [21, 24, 27–30, 32, 34]. It included three groups, socio-demographic, pregnancy related, and psychosocial factors.

### 3.3 Pooled prevalence of khat chewing among pregnant women in Ethiopia

**Fig 2:**
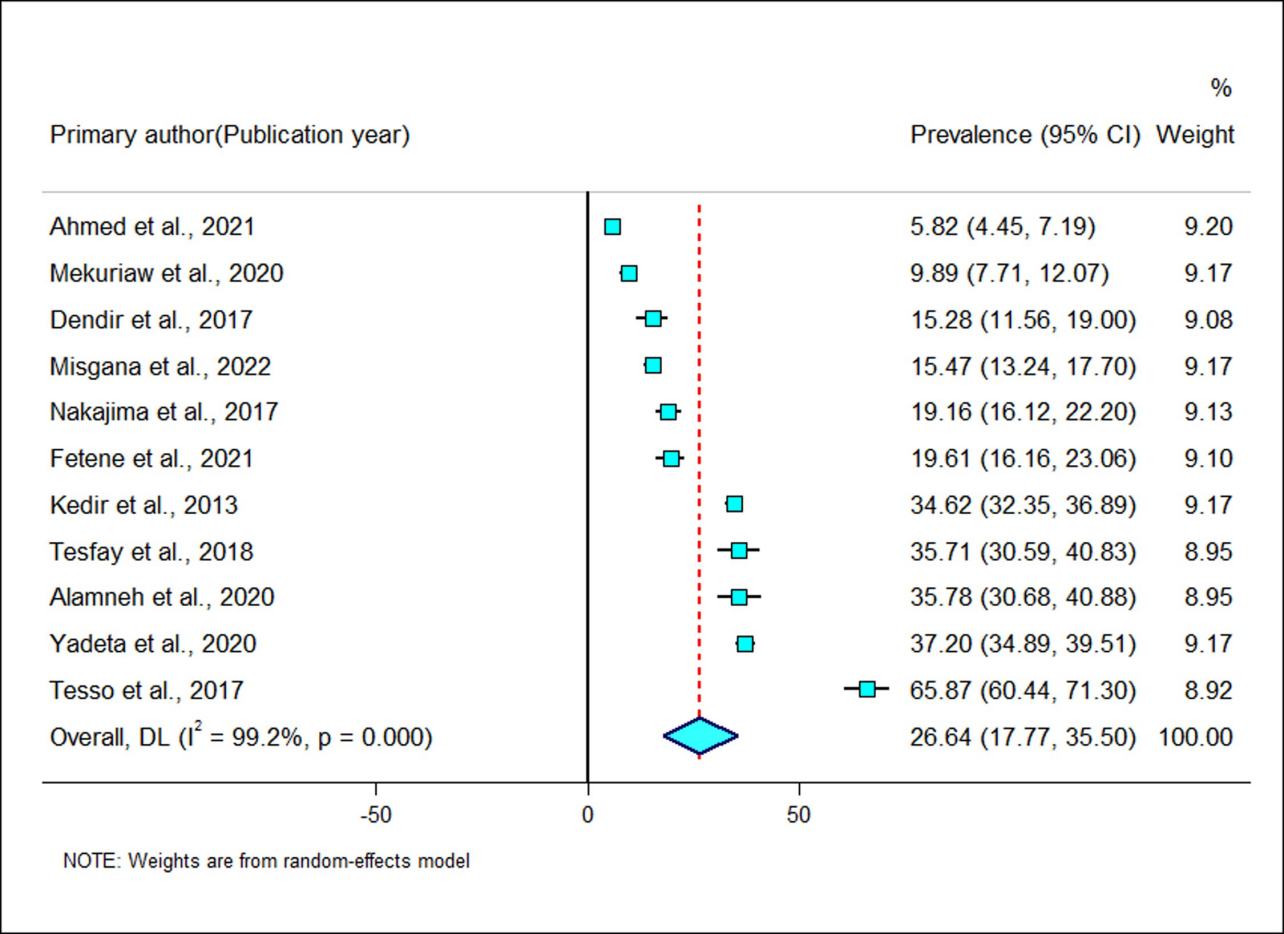
Forest plot of the prevalence of khat use among pregnant women in Ethiopia.

The khat chewing prevalence in the 11 studies included in the meta-analysis ranged from 5.82% in Jimma town [19] to 65.9% in Jimma town [20]. Out of the 11 studies, only two studies reported prevalence of less than 10% [19, 39]. In accordance with the random effects model analysis, the pooled prevalence of khat chewing among pregnant women in the 11 studies was 26.6% (95%CI 17.8–35.5), with heterogeneity index (I^2^) of 99.2% (*p-value*<0.001) (Fig 2), confirming significant variability among studies.

### 3.4 Pooled prevalence of alcohol use among pregnant women in Ethiopia

**Fig 3:**
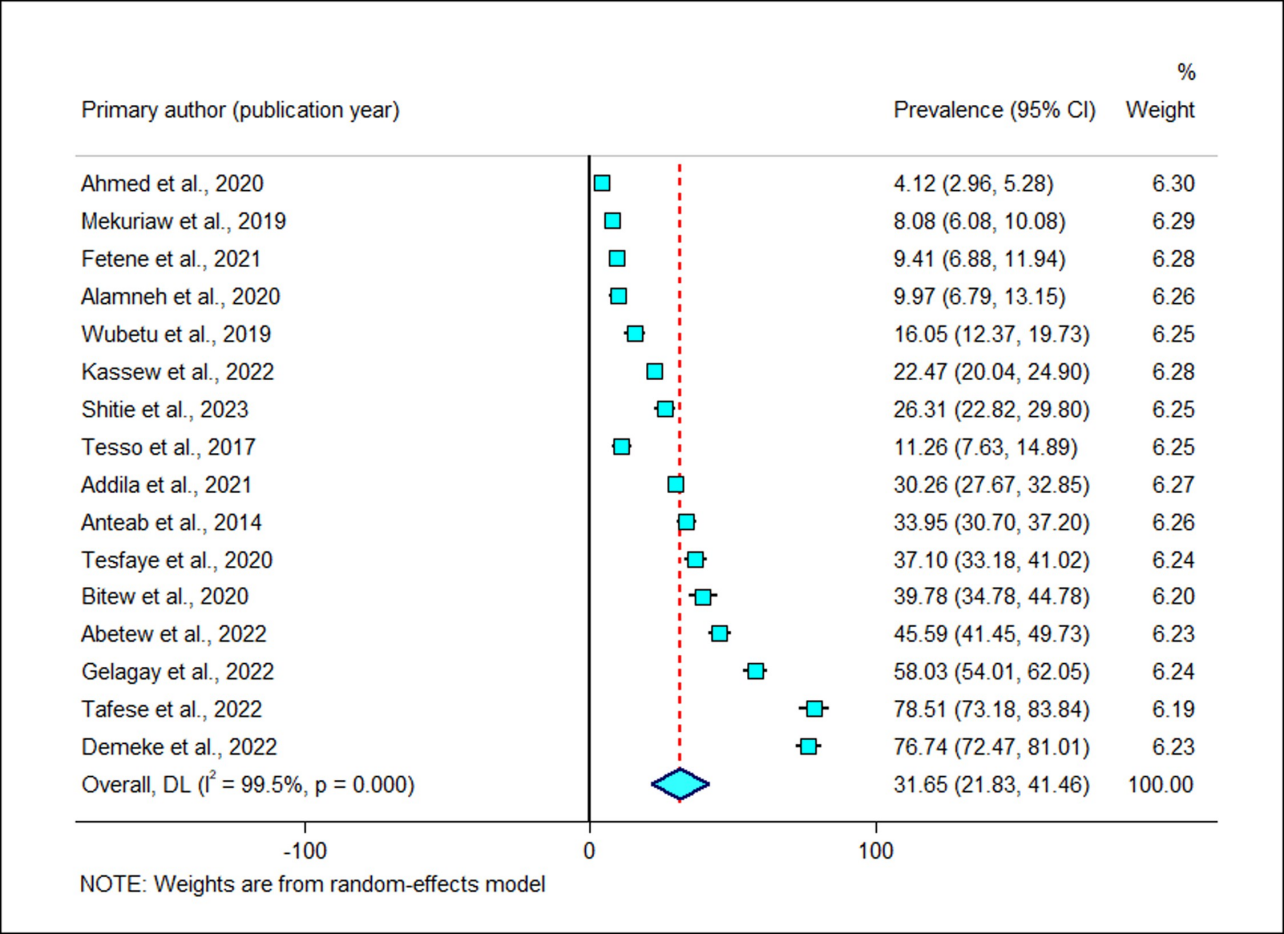
Forest plot of the prevalence of alcohol use among pregnant women in Ethiopia.

Sixteen studies [19–34] had reported the prevalence of alcohol consumption among pregnant women. The reported prevalence of alcohol consumption among pregnant women among studies included in this meta-analysis ranges from 4.1% in Oromia [19] to 76.7% in Amhara region [34]. The pooled prevalence of alcohol consumption among pregnant women using the random effect model was found to be 31.6% (95% CI: 21.8, 41.5). There was significant variability among studies (I^2^ = 99.5% and *p*-value< 0.001) (Fig 3).

### 3.5 Subgroup analysis

**Fig 4:**
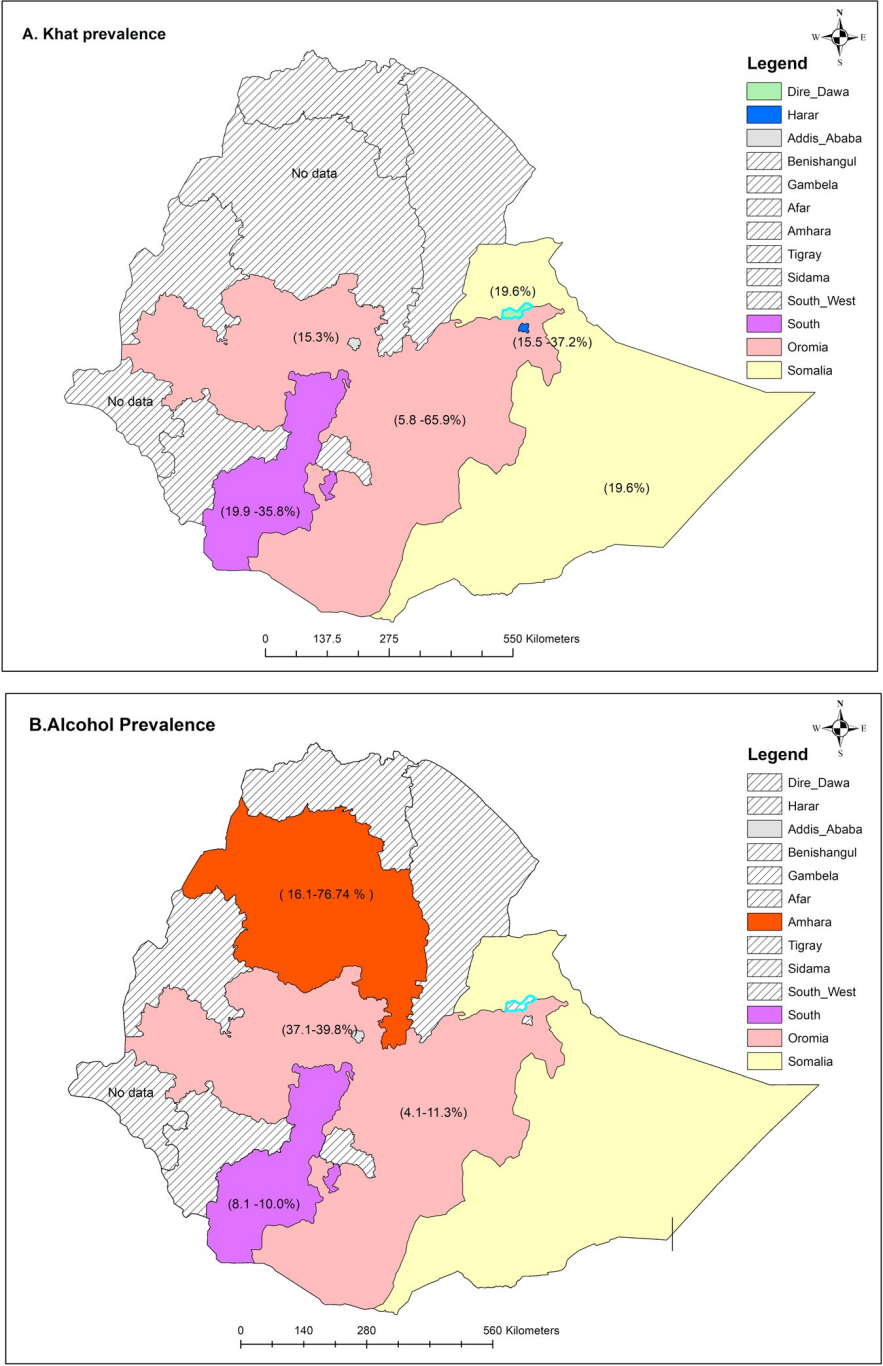
A and B. Regional distribution of khat and alcohol use among pregnant women in Ethiopia. Source: The shape file accessed from Humanitarian Data Exchange website (https://data.humdata.org/dataset/cod-ab-eth).The shapefile is public domain and can be used freely.

S4A Table presents the findings of the sub-group analysis of the pooled prevalence of khat chewing, including evaluation of variations between sub-groups. The sub-group analysis by region showed that the Oromia region had the highest khat prevalence (31.5%, 95%CI 9.7–53.3) while Addis Ababa city had the lowest prevalence (15.3%, 95%CI 11.6–19.0) (Fig 4A). In reference to the study settings, the prevalence was higher in community-based studies (28.5%, 95%CI 14.1–42.9) than in health facility-based studies (25.9%, 95%CI 14.7–37.1). The prevalence of khat chewing differs significantly between studies conducted in community and health facility settings (*p*<0.001). The results of the subgroup analysis also revealed that the pooled prevalence was higher in studies included a sample of less than 790 pregnant women (28.6%, 95%CI 16.9–40.2).This difference was statistically significant (*p*<0.01). According to the findings, the study area, study setting, and sample size were the sources of heterogeneity.

S4B Table shows the subgroup analyses of alcohol consumption prevalence among pregnant women based on the study region, study setting, data collection tool, sample size and mean age. The subgroup analysis by region showed that the Amhara region had the highest prevalence (45.6%, 95% CI: 31.2, 60.0) while the South region had the lowest prevalence (8.6%, 95% CI: 6.9, 10.3) (Fig 4B).

When stratified by study setting, the prevalence of alcohol use was greater in community-based studies (44.4%, 95% CI: 29.2, 59.6) than in institution-based studies (18.9%, 95% CI: 10.9, 26.8). In reference to the data collection tool, studies conducted using CAGE accounted for the lowest prevalence estimate of 16.0% (95% CI: 12.4, 19.7), compared with dichotomous question 35.57% (95%CI: 19.8, 51.3). We classified the studies into 2 groups according to the mean age of respondents (< 30 and ≥30 years of age) and found that the prevalence of alcohol use was higher among pregnant women with a mean age of ≥30 years (71.0; 95% CI: 57.6, 84.4) than those < 30 years of age (25.6; 95% CI: 15.2, 29.9). This variation was statistically significant (*p*< 0.001).The sub-group analysis based on sample size showed that the prevalence of alcohol consumption was 37.1% (95% CI: 22.8, 51.44) for studies that included a sample of less than 613 pregnant women, and it was 19.7(95% CI; 8.1, 31.4) for studies that included a sample of 613 and above pregnant women. This difference was statistically significant (*p*<0.001). According to the findings, the study region, study setting, sample size, data collection tool, and mean age were the sources of heterogeneity for alcohol prevalence (S4B Table).

### 3.6 Publication bias

A funnel plot and the Eggers test were used to assess publication bias. The funnel plot of the articles included in the meta-analysis reveals no publication bias for prevalence of khat chewing among pregnant women. The absence of publication bias was further confirmed by the Eggers test (*p* = 0.142). Egger’s tests p-value was (*p*  =  0.287) showing the absence of publication bias for the prevalence of alcohol consumption among pregnant women (S1 Fig).

### 3.7 Sensitivity analysis

For khat and alcohol use, we examined the source of heterogeneity further by performing a leave-one-out sensitivity analysis. The sensitivity analysis revealed that when each study was excluded from the analysis, the average estimated prevalence of khat chewing obtained was within the 95% CI of the average prevalence of khat chewing obtained when all studies were run together. As a result, the average prevalence of khat chewing among pregnant women was not affected by a single study. Furthermore, the sensitivity findings indicate that when each study was omitted, the average khat chewing prevalence ranged between 22.8% (95% CI: 14.6, 30.9) and 27.7% (95% CI: 20.2, 37.3) (S5A Table). The results of the sensitivity analysis also showed that the pooled estimate of alcohol consumption was not dependent on a single study. The 95% CI of the pooled prevalence of alcohol consumption overlapped after omitting a single study in the sensitivity analysis but varied between 28.5% (95% CI; 19.2, 37.9) and 33.5% (95% CI; 23.5, 43.5) (S5B Table).

### 3.8 Meta-regression

There was considerable heterogeneity among the studies included in the meta-analysis. To investigate the origins of heterogeneity, we conducted a meta-regression analysis using region, study period, sample size, mean age, data collection tool, and study setting. None of the variables in the meta-regression analysis had a significant influence on the observed difference in khat chewing prevalence among pregnant women in Ethiopia (S6A Table).

**Fig 5:**
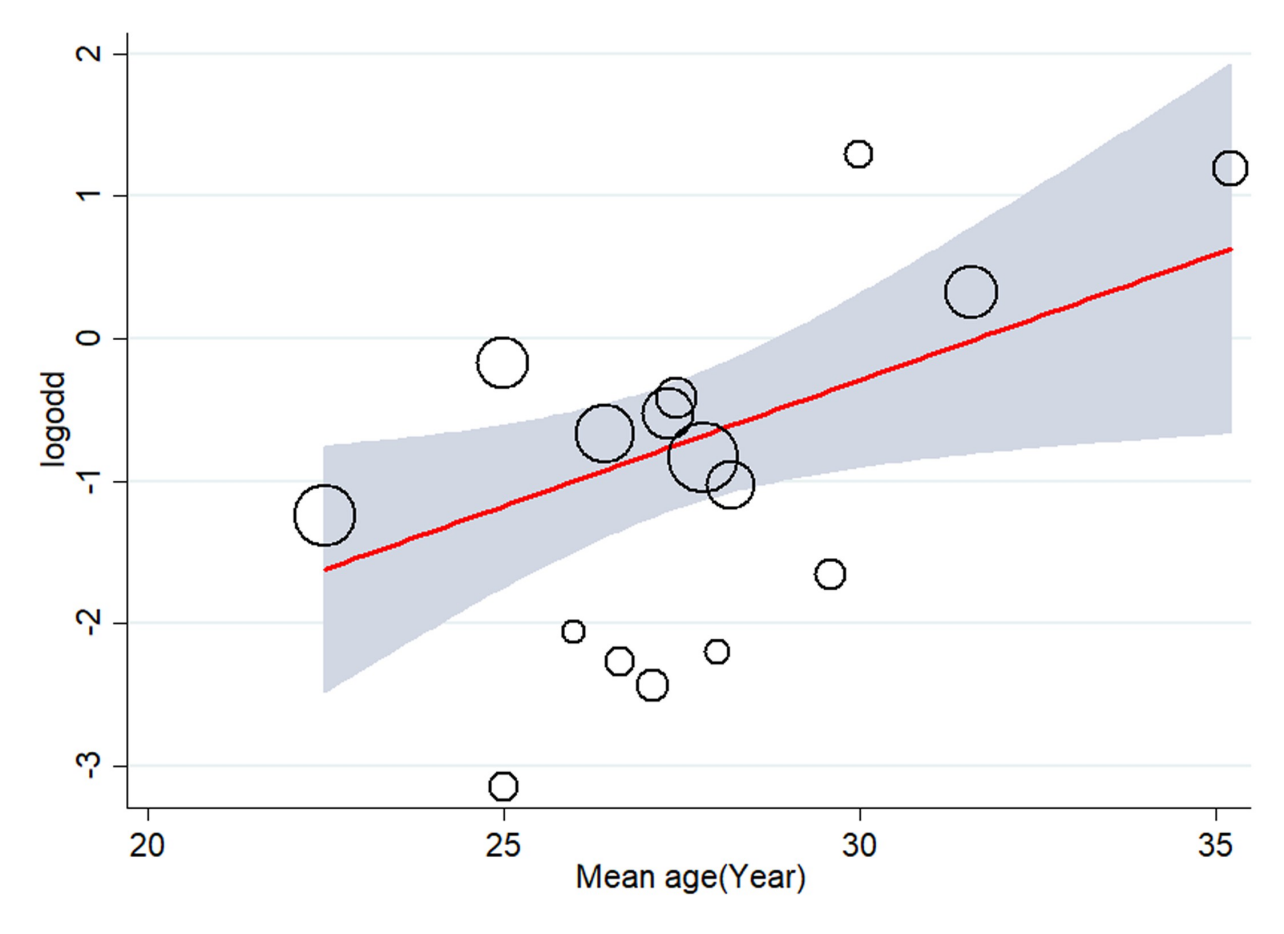
Meta-regression of alcohol use against mean age.

The results of the meta-regression analysis also revealed that the prevalence of alcohol consumption was lower in studies conducted in the Oromia region (β  = -2.138, 95% CI: -3.942, -0.333, *p-value* = 0.020), and South region (β  = -1.845, 95% CI: -3.648, -0.043, *p-value* = 0.045) as compared to Addis Ababa. When we set health facility as the reference study setting, studies conducted in community setting showed higher prevalence of alcohol consumption (β  = 1.399, 95% CI: 0.335, 2.464, *p-value* = 0.010). The meta-regression analysis revealed that there was no significant difference by study period (*p-value* = 0.192), sample size (*p-value* = 0.208) and data collection tool (*p* = 0.701 and *p* = 0.723). The result of the meta-regression analysis also showed that the prevalence of alcohol consumption increased with a mean age (β  =  0.258, 95% CI: 0.075, 0.441, *p*<0.01) (S6B Table). Similarly, the bubble plot figure showed that the prevalence of alcohol intake increased with increasing mean age (Fig 5).

### 3.9 Factors associated with khat chewing among pregnant women

**Fig 6:**
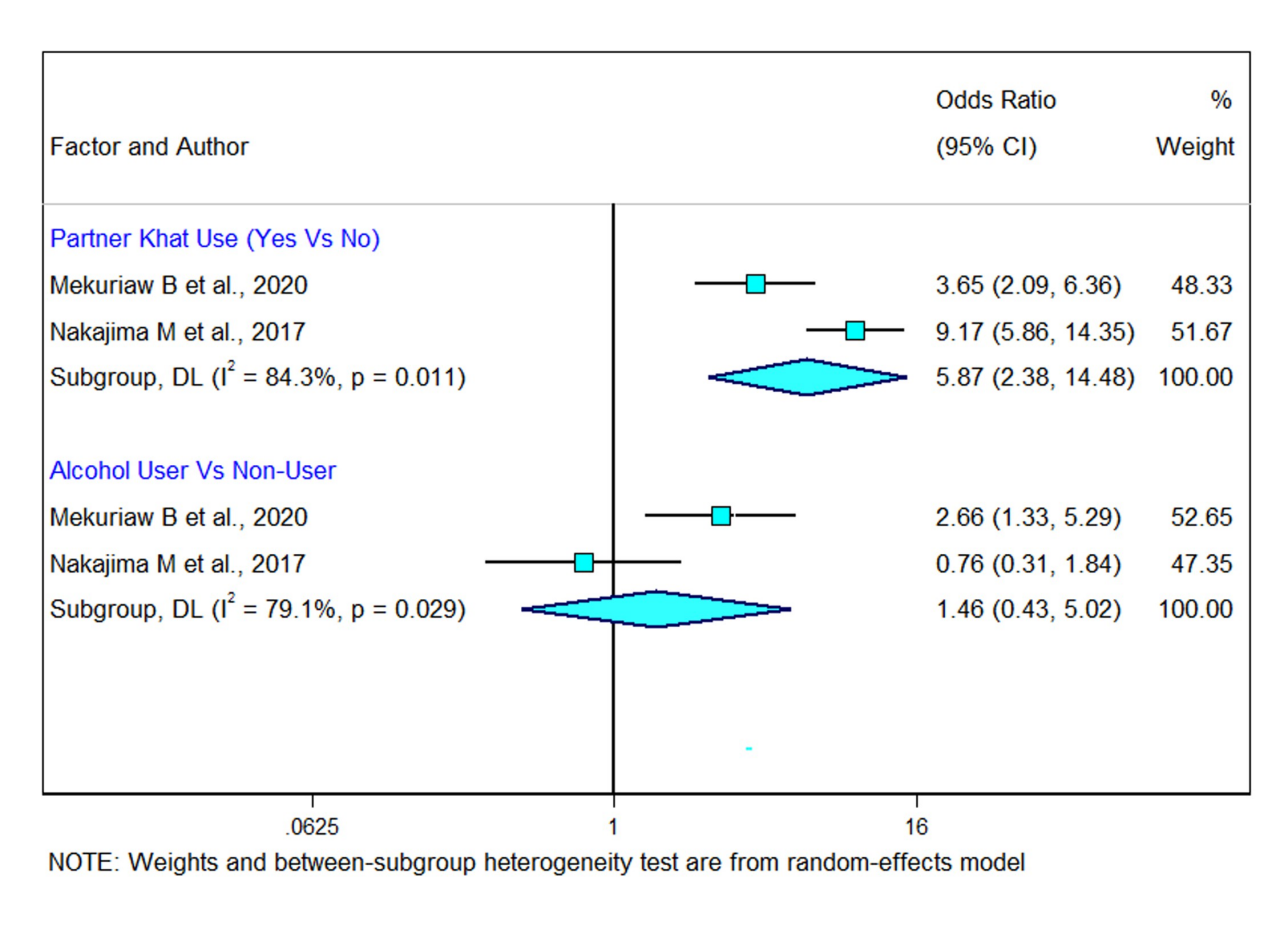
Forest plot showing factors associated with khat use during pregnancy in Ethiopia.

Two studies out of the total twelve studies included in this meta-analysis reported on factors associated with khat chewing. Two studies indicated that pregnant women with a khat chewer partner were more likely than women without a khat chewer partner to consume khat during pregnancy [39, 41]. The combined result based on the two studies showed an overall estimate OR of partner khat use as a determinant of khat chewing during pregnancy was 5.87 (95% CI 2.38, 14.48; I^2^ = 84.3%; *p-value* = 0·011). Only one study show that the odds of current khat chewing were 2.66 times (OR 2.66 [95% CI (1.33, 5.29)]) higher among pregnant women who consumed alcohol compared to those who did not consume alcohol [39]. The pooled odds ratio of alcohol use among these studies was found to be 1.46 (95% CI: 0.43, 5.02) (I^2^ =  79.1%, *p*<0.05). This suggested that there was no significant association between alcohol consumption and khat chewing during pregnancy (Fig 6).

### 3.10 Factors associated with alcohol use among pregnant women

In this systematic review and meta-analysis, some of the factors associated with alcohol use among pregnant women were pooled quantitatively and some were not because of inconsistent classification of the independent variables to the dependent variable. Thus, those determinants consistently reported in more than one original study were included in this meta-analysis.

#### 3.10.1 Socio-demographic factors

**Fig 7:**
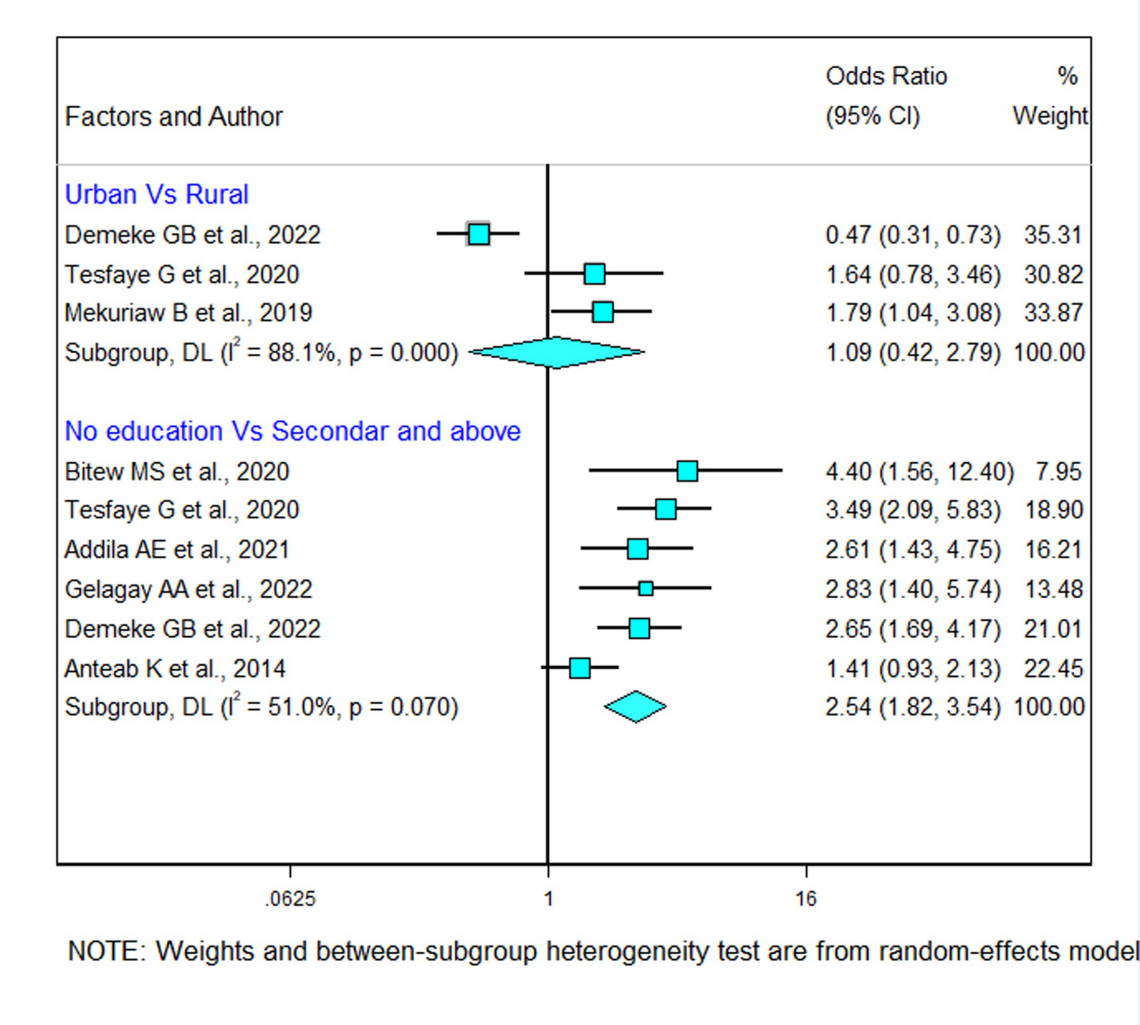
Forest plot showing the association between socio-demographic factors and alcohol use during pregnancy in Ethiopia.

Socio-demographic characteristic factors were examined in six studies. Six studies were included to assess the association between level of education and alcohol use during pregnancy [27–30, 32, 34]. Five studies found a significant association between decreased educational level and alcohol consumption during pregnancy [27, 29, 30, 32, 34]. The pooled odds ratio showed that non-educated pregnant women were 2.54 times more likely to use alcohol than those who completed college and above educational level (OR 2.54 [95% CI (1.8, 3.5)]). There is a moderate heterogeneity across the studies (I^2^ = 51.0% and *p*-value = 0.070). One study indicated that pregnant women who residing in urban areas were more likely to use alcohol as compared to those residing in rural areas [21]. However, the pooled odds ratio showed that there was no significant association between residence and alcohol consumption (OR 1.09 [95% CI (0.4, 2.8)]) (I^2^ =  88.1%, *P* < 0.01) (Fig 7).

#### 3.10.2 Pregnancy related factors

**Fig 8:**
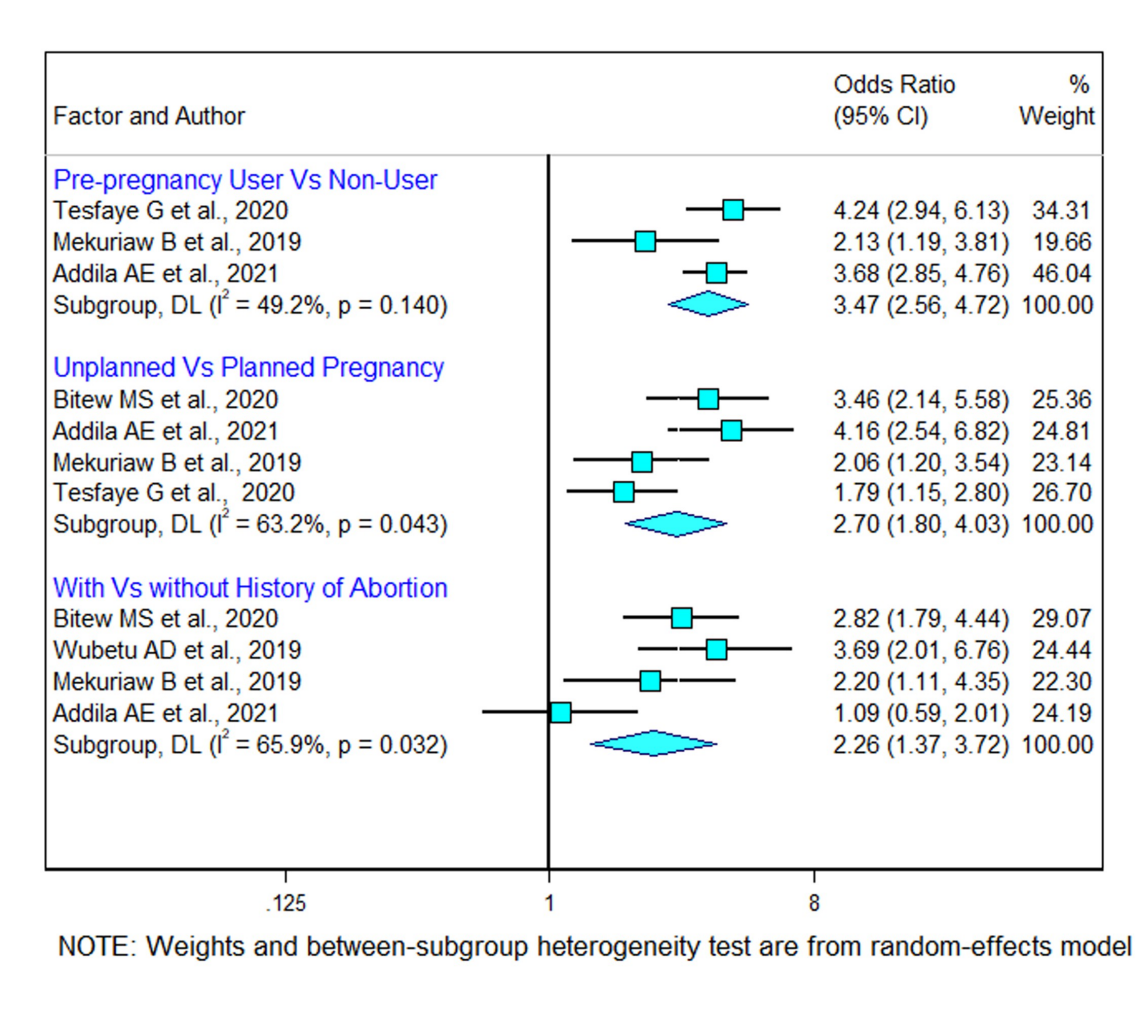
Forest plot showing the association between pregnancy related factors and alcohol use during pregnancy in Ethiopia.

The meta-analysis of pregnancy related factors was based on five cross-sectional studies. Three studies indicated that pre-pregnancy alcohol consumption was significantly associated with alcohol use during pregnancy [21, 27, 29]. According to the pooled odds ratio, pregnant women who reported consuming alcohol before to pregnancy were more than three times (OR 3.5 [95% CI (2.6, 4.7)]) more likely to drink alcohol during pregnancy than women who did not have a previous history of alcohol consumption. There was no significant heterogeneity across the included studies (I^2^ = 49.2% and *p*-value = 0.140). A total of four studies were included in this meta-analysis to show the significance between pregnancy plan and alcohol consumption. The pooled odds ratio of unplanned pregnancy in these studies was found to be 2.7 (95% CI: 1.8, 4.0). This finding suggested that women whose last pregnancy was unplanned were 2.70 times more likely to consume alcohol during pregnancy than their counters. There is a moderate heterogeneity across the studies (I^2^ = 63.2% and *p*-*value* < 0.05). Four studies were included to assess the association between history abortion and alcohol use among pregnant women [21, 24, 27, 30]. The findings showed that the history of abortion was significantly associated with alcohol use among pregnant women. The pooled odds ratio showed that pregnant women that reported having a history of abortion were two times more likely to consume alcohol as compared with those that did not report any abortion history (OR 2.26 [95% CI (1.37, 3.72)]). There is a moderate heterogeneity (I^2^ = 65.9% and *p*-value = 0.032) was observed across the included studies (Fig 8).

#### 3.10.3 Psychosocial factors

**Fig 9:**
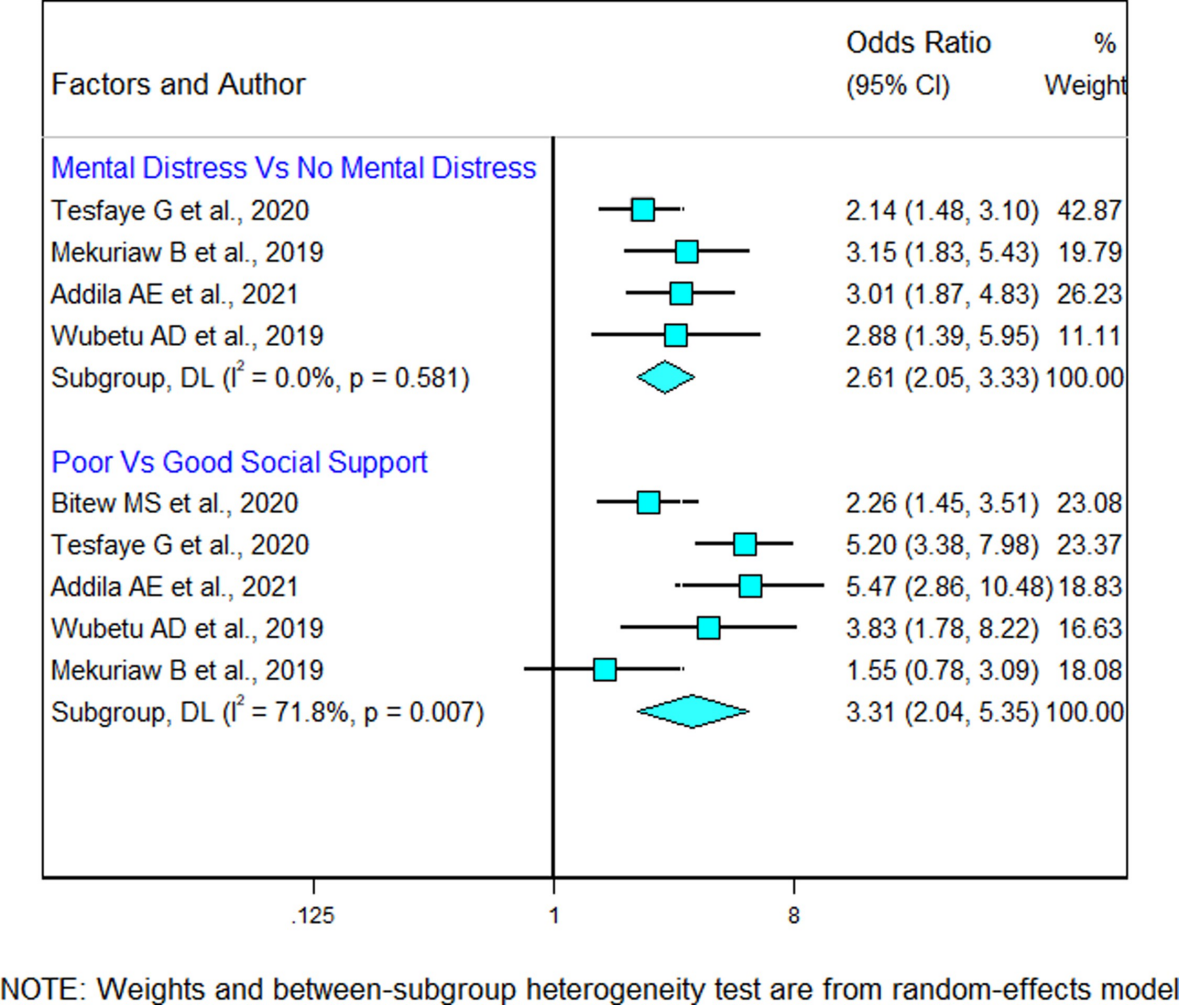
Forest plot showing the association between psychosocial factors and alcohol use during pregnancy in Ethiopia.

Five studies were included to assess the association between psychosocial factors and alcohol use during pregnancy [21, 24, 27, 29, 30]. All studies showed that social support was significantly associated with alcohol use during pregnancy. The average odds ratio of poor social support in these studies was found to be 3.31 (95%CI: 2.0, 5.3). This result suggested that pregnant women with poor social support were 3 times more likely to use alcohol during pregnancy as compared to those with good social support. Likewise, four of the above-indicated studies [21, 24, 27, 29] had also reported mental distress as a risk factor for alcohol use during pregnancy. The pooled adjusted odds ratio of mental distress among these studies was found to be 2.61 (95% CI: 2.0, 3.3). There was no heterogeneity across the included studies (I^2^ = 0% and *p*-value = 0.581) (Fig 9).

## 4. Discussion

The primary objective of this research was to conduct an updated evidence synthesis of the prevalence and risk factors of khat chewing and alcohol use among pregnant women by systematic means of identifying, selecting and critically appraising the literatures. This work is, to the best of our knowledge, the first comprehensive and detailed review of prevalence and risk factors of khat and alcohol use among pregnant women in Ethiopia.

The current meta-analysis revealed that the pooled prevalence of khat chewing among pregnant women in Ethiopia was 26.6%. The pooled prevalence of khat chewing in the current review was lower as compared to the result of a study done in Yemen [37]; which reported the prevalence of khat chewing among pregnant women to be 40.7%. This variation might be explained by differences in social and cultural factors. Khat is the national plant and a daily habit in Yemen. Additionally, Yemen is a Muslim-majority country. There is an important connection between Muslims and khat use, and it is stated that Muslims are the world’s largest khat consumers.

In our subgroup analysis, the prevalence of khat chewing varied greatly in different regions in Ethiopia and ranged from 15.3% to 31.5% with the highest prevalence reported in the Harar and Oromia region. The region difference might be attributed to differences in sociocultural elements and availability of khat farms in various locations. In particular, Harar region is regarded to be the origin of khat and the primary historical location for its consumption, a tradition with profound historical origins [45]. Furthermore, Muslims constitute about 47.5% and 69.0% of the population in Harar and Oromia region, respectively [46]. Evidence suggests a high association between Muslims and khat consumption. Many Muslims in Ethiopia chew khat while visiting pilgrimage sites and performing rituals such as singing, praying (du’a), blessing, and other practices. Muslim women also use khat when they assemble for Fatimaye, a social ritual honoring the name of Fatima, Prophet Muhammad’s daughter, to pray for mothers in labor [47].

The pooled prevalence of alcohol use among pregnant women in Ethiopia was found to be 31.6%. This revealed that three out of every ten pregnant women consume alcohol. This figure, however, is nearly twofold higher than that of certain developed countries [48–50]. This implies that alcohol use during pregnancy is a major public health issue in Ethiopia that requires attention and intervention from ministry of health. A study conducted in Sweden showed that only 12% of pregnant women use alcohol [49]. Similarly, another study conducted among 5882 pregnant women in Canada reported that 10.8% of them use alcohol [50]. This variance might be explained by differences in access to antenatal care services and awareness of prenatal care service utilization. In Ethiopia, a large majority of pregnant women do not obtain proper antenatal care (ANC) [51]. As a result, insufficient ANC results in missed screening of alcohol use. Moreover, the finding of the present review is higher than a WHO estimate of alcohol consumption during pregnancy in Ethiopia (7.9%) [17], meta-analysis in sub-Saharan Africa (20.8%) [18], Tanzania (21.2%) [52], and demographic health survey in sub-Saharan Africa (22.8%) [53]. The disparity in prevalence between this study and the WHO review may be explained in part by variations in methodology, since our review was a meta-analysis that included 17 studies to estimate prevalence alcohol use among pregnant women in Ethiopia, but the WHO review only used one study. However, the current study result is lower than a study done in South Africa (36.9%) [54], Ghana (48%) [55] and Nigeria (59.3%) [56].

Alcohol prevalence estimates differed by region, as indicated by subgroup analysis. The highest 45.6% and the least prevalence of 7.5% were obtained from Amhara and Oromia regions, respectively. This is consistent with the pattern observed earlier in the EDHS 2016 report [25]. This finding implies that there is regional difference in the prevalence of alcohol consumption among pregnant women in Ethiopia, which has to be further investigated in order for policymakers to direct more resources to areas where the prevalence is high.

The effect sizes of associated factors for khat chewing in pregnant women were estimated in this review. Women whose partners used khat were more likely to use khat during pregnancy than women whose partners did not use khat. This result is consistent with a study conducted in Yemen [57]. This might be explained by the fact that pregnant women are easily influenced by their husbands to use khat when pregnant. Men are an important stakeholder and should be regarded half of the equation in mother and child health [58].

In this study, we also reviewed studies that looked at risk factors for alcohol use during pregnancy and classified the numerous related factors into three categories: (a) socio-demographic factors, (b) pregnancy-related factors, and (d) psychosocial factors. In regard of women’s educational levels, illiterate women have a higher positive link with alcohol use during pregnancy than educated women. The explanation for this significant association might be that educated women are aware of the risks of drinking alcohol during pregnancy [59]. However, this is contrary to findings from studies in Netherland [60] and Japan [61].

Social support was another factor that was significantly associated with alcohol use during pregnancy in this study. Women with poor social support were three times more likely to drink alcohol during pregnancy than those with good social support. This finding is consistent with the study conducted in Sweden [62]. The likely reason for the association between social support and alcohol use is that when pregnant women have strong social support, they are more likely to share information about their pregnancy and other maternal health services, which can help shape women’s health-seeking behavior. Additionally, we observed that alcohol use during pregnancy was associated with mental distress. In agreement with a result of a systematic review conducted in Sub-Saharan Africa [18] pregnant women with mental distress were 2.46 times more likely to use alcohol as compared to pregnant women without mental distress. This could be because people in mental distress are more inclined to consume alcohol to overcome the challenges associated with sleep initiation, social interaction, and a lack of satisfaction, all of which are typical symptoms of mental distress [63].

Unplanned pregnancy has also been linked to an increase in alcohol usage among pregnant women in Ethiopia. This result is consistent with result found in Korea [64].The probable rationale might be the social and psychological instability of an unanticipated pregnancy, which has the ability to motivate pregnant women to begin using alcohol or other substances to cope with stress [65]. Our review also revealed that pre-pregnancy alcohol use was significantly associated with alcohol consumption during pregnancy. This finding was consistent with the finding of studies conducted in Sweden [62] and Australia [66]. One meta-analysis found that, preconception counseling can result in a significant reduction in alcohol consumption during the first trimester [67]. Therefore, a wide diversity of sectors and stakeholders must be involved to ensure full access to preconception care. Additionally, all women of reproductive age should be screened for alcohol consumption and educated about the possible dangers of alcohol consumption during pregnancy to the fetus.

### 4.1 Implications of the findings

The current study is different from the previous systematic review and meta-analysis [35] in that it estimated the pooled odds ratio of risk factors associated with alcohol use during pregnancy. It also estimated the pooled prevalence based on geographical locations. The results of our review may help government officials, policymakers, researchers, and other stakeholders identify the regions in Ethiopia with a high prevalence of khat and alcohol use during pregnancy. Our review revealed that there was a regional difference in the prevalence of alcohol consumption during pregnancy in Ethiopia, which has to be further investigated in order for policymakers to direct more resources to areas where the prevalence is high. Attention needs to be given to substance use during pregnancy, and the government needs to consider the possible integration of Alcohol, Smoking and Substance Involvement Screening Test (ASSIST) linked-brief interventions with the existing ANC in Ethiopia. This review also provides the pooled prevalence of khat and alcohol use during pregnancy, which can help determine whether Ethiopia is making progress towards achieving Sustainable Development Goal (SDG) 3 (targets 3.1, 3.2, and 3.5) by 2030.

### 4.2 Literature gaps

There are several aspects of khat and alcohol consumption during pregnancy that should be investigated. First, there were some regions that were not covered by the studies included in this review and meta-analysis.

Second, the influence of community-level factors such as the density of khat or alcohol outlets per area on khat or alcohol use was not explored. Third, there is insufficient research on the interaction effects of socio-demographics and obstetric factors. Fourth, none of the research used validated data collection tools to measure khat addiction level during pregnancy. Finally, there is a scarcity of prospective cohort studies that assess patterns of khat and alcohol consumption throughout pregnancy.

### 4.3 Strengths and limitations of the review

This review has several strengths, including the fact that it is the first review to estimate the pooled odds ratio of a broad range of potential risk factors associated with khat and alcohol use among Ethiopian pregnant women. During the evaluation process, we also followed the PRISMA 2020 guidelines. Furthermore, the majority of the studies in this analysis had response rates higher than 85%, indicating that the review had a minimal risk of attrition bias. Because of the following methodological limitations, the findings of this review should be considered with care. To begin, due to insufficient primary studies, the review was based on studies conducted in six of the country’s 10 regions, limiting the generalizability of the findings at the national level. Second, all include studies that gathered data on khat and alcohol use using self-reported measures, but no study collected biochemical data. Self-reported consumption may be influenced by recall and self-declaration bias, resulting in under- or over-reporting of the outcome of interest. As a result, the real prevalence of khat and alcohol consumption among pregnant women may be underestimated or overestimated. Finally, heterogeneity persisted even in certain subgroup analysis, which is unavoidable in epidemiological meta-analysis [68].

## 5. Conclusions

Our results show that the pooled prevalence of khat chewing and alcohol use among pregnant women in Ethiopia is high and is unevenly distributed among various regions. There is an urgent need for targeted interventions for groups of pregnant women with a particular high risk of alcohol use, such as illiterate women, pre-pregnancy alcohol use, unplanned pregnancy, women with history of abortion, women with poor social support, and mental distress. The influence of community-level factors such as the density of khat or alcohol outlets per area on khat or alcohol use was not explored. Studies closing this knowledge gap are therefore required. Effective substance use screening in all women of childbearing age, as well as preconception health promotion, should become a standard of care in all health care settings. Women who have been identified as having a substance use disorder should be referred to substance use disorder treatment centers. Social marketing campaigns and point-of-sale warnings in local languages targeting women of reproductive age are essential to reduce the burden.

## Supporting information

S1 Table PRISMA 2020 check list. (DOCX)

S2 Table Search strategy for khat chewing, and alcohol drinking prevalence during pregnancy studies from African countries. (DOCX)

S3 Table A and B. Study characteristics included in the systematic review and meta-analysis on prevalence of khat and alcohol use among pregnant women in Ethiopia.
(ZIP)

S4 Table A and B. Sub-group analysis of khat and alcohol use by different characteristics in Ethiopia.
(ZIP)

S5 Table A and B. Sensitivity analysis showing presence of influential study among studies conducted to determine khat and alcohol prevalence among pregnant women in Ethiopia.
(ZIP)

S6 Table A and B. Meta-regressions of khat use among pregnant women in Ethiopia by sample size, and publication year of included studies.
(ZIP)

S1 Fig Funnel plot and egger test for pooled prevalence of khat and alcohol use among pregnant women in Ethiopia. (TIF)
