# Supplementary material for: The epidemiology of khat (catha edulis) chewing and alcohol consumption among pregnant women in Ethiopia: A systematic review and meta-analysis
Source: PLOS Glob Public Health. 2023 Sep 15;3(9):e0002248. doi: 10.1371/journal.pgph.0002248 (PMC10503716; doi:10.1371/journal.pgph.0002248)
Supplement: S2 Table — (DOCX) [file pgph.0002248.s002.docx]

S2 Table Search strategy for prenatal khat chewing and alcohol drinking prevalence studies from Ethiopia

| Database I: PubMed (n=121) | |
| --- | --- |
| Sequence | Query |
| #1 | (((((((((khat*[MeSH Terms]) OR (khat*[Title/Abstract])) OR (catha*[MeSH Terms])) OR (catha*[Title/Abstract])) OR (catha edulis[MeSH Terms])) OR (catha edulis[Title/Abstract])) OR (qat plant*[MeSH Terms])) OR (qat plant*[Title/Abstract])) OR (miraa*[MeSH Terms])) OR (miraa*[Title/Abstract]) |
| #2 | (((((((((alcohol[MeSH Terms]) OR (alcohol[Title/Abstract])) OR (alcohol abuse[MeSH Terms])) OR (alcohol abuse[Title/Abstract])) OR (alcohol drinking[MeSH Terms])) OR (alcohol drinking[Title/Abstract])) OR (alcohol consumption[MeSH Terms])) OR (alcohol consumption[Title/Abstract])) OR (binge drinking[MeSH Terms])) OR (binge drinking[Title/Abstract]) |
| #3 | (((substance use disorder*[MeSH Terms]) OR (substance use disorder*[Title/Abstract])) OR (substance abuse[MeSH Terms])) OR (substance abuse[Title/Abstract]) |
| #4 | #1 OR #2 OR #3 |
| #5 | (((((((pregnant women[MeSH Terms]) OR (pregnant women[Title/Abstract])) OR (pregnant woman[MeSH Terms])) OR (pregnant woman[Title/Abstract])) OR (pregnancy[MeSH Terms])) OR (pregnancy[Title/Abstract])) OR (prenatal care[MeSH Terms])) OR (prenatal care[Title/Abstract]) |
| #6 | (Ethiopia[MeSH Terms]) OR (Ethiopia[Title/Abstract]) |
| #7 | #4 AND #5 AND #6  ((((((((((((khat*[MeSH Terms]) OR (khat*[Title/Abstract])) OR (catha*[MeSH Terms])) OR (catha*[Title/Abstract])) OR (catha edulis[MeSH Terms])) OR (catha edulis[Title/Abstract])) OR (qat plant*[MeSH Terms])) OR (qat plant*[Title/Abstract])) OR (miraa*[MeSH Terms])) OR (miraa*[Title/Abstract])) OR ((((((((((alcohol[MeSH Terms]) OR (alcohol[Title/Abstract])) OR (alcohol abuse[MeSH Terms])) OR (alcohol abuse[Title/Abstract])) OR (alcohol drinking[MeSH Terms])) OR (alcohol drinking[Title/Abstract])) OR (alcohol consumption[MeSH Terms])) OR (alcohol consumption[Title/Abstract])) OR (binge drinking[MeSH Terms])) OR (binge drinking[Title/Abstract]))) AND ((((((((pregnant women[MeSH Terms]) OR (pregnant women[Title/Abstract])) OR (pregnant woman[MeSH Terms])) OR (pregnant woman[Title/Abstract])) OR (pregnancy[MeSH Terms])) OR (pregnancy[Title/Abstract])) OR (prenatal care[MeSH Terms])) OR (prenatal care[Title/Abstract]))) AND ((Ethiopia[MeSH Terms]) OR (Ethiopia[Title/Abstract])) |
| Database II: Cochrane Library (n=12) | |
| Sequence | Query |
| #1 | (“khat”):ti,ab,kw OR (“Catha edulis”):ti,ab,kw OR (“qat”):ti,ab,kw (Word variation has been searched) |
| #2 | (alcohol):ti,ab,kw OR (alcohol abuse):ti,ab,kw OR (alcohol drinking):ti,ab,kw OR (binge drinking):ti,ab,kw OR (Word variation has been searched) |
| #3 | (substance use):ti,ab,kw OR (substance abuse): ti,ab,kw (Word variation has been searched) |
| #4 | #1 OR #2 OR #3 |
| #5 | (pregnant):ti,ab,kw OR (pregnancy):ti,ab,kw OR (Word variation has been searched) |
| #6 | (Ethiopia):ti,ab,kw (Word variation has been searched) |
| #7 | #4 AND #5 AND #6 |
| Database III: Science direct (n=730) | |
| Sequence | Query |
| #1 | (Khat OR "Catha edulis" OR alcohol OR "Substance use" OR "Substance abuse") AND (pregnant OR pregnancy) AND (Ethiopia) |
| Database IV: Google scholar (n=20) | |
| Sequence | Query |
| #1 | allintitle: (khat OR alcohol OR substance use) AND (pregnancy OR pregnant) AND (Ethiopia) |
| Database V: African Journal Online (n=76) | |
| Sequence | Query |
| #1 | khat alcohol pregnant Ethiopia |
| Database VI:WHO African Index Medicus (n=2) | |
| Sequence | Query |
| #1 | (tw:((Khat OR alcohol) AND (Pregnant))) AND (tw:(Ethiopia)) |
