# Supplementary material for: The epidemiology of khat (catha edulis) chewing and alcohol consumption among pregnant women in Ethiopia: A systematic review and meta-analysis
Source: PLOS Glob Public Health. 2023 Sep 15;3(9):e0002248. doi: 10.1371/journal.pgph.0002248 (PMC10503716; doi:10.1371/journal.pgph.0002248)
Supplement: S3 Table — A and B. Study characteristics included in the systematic review and meta-analysis on prevalence of khat and alcohol use among pregnant women in Ethiopia. (ZIP) [file pgph.0002248.s003.zip › S3A_Table.docx]

**S3A Table**. Characteristics of studies included in meta-analysis of khat chewing among pregnant women in Ethiopia.

| **S.No.** | **Author** | **Publication year** | **Region** | **Study setting** | **Study design** | **Sample size** | **Numb chewers** | **Study period** | **Prevalence (%)** |
| --- | --- | --- | --- | --- | --- | --- | --- | --- | --- |
| 1 | Ahmed et al | 2021 | Oromia | Health facility | Cross sectional | 1117 | 65 | 2017 | 5.82 |
| 2 | Mekuriaw et al | 2020 | SNNPR | Health facility | Cross sectional | 718 | 71 | 2017 | 9.89 |
| 3 | Dendir et al. | 2017 | Addis Ababa | Health facility | Case control | 360 | 55 | 2016 | 15.28 |
| 4 | Misgana et al. | 2022 | Eastern Ethiopia | Community | Cross sectional | 1015 | 157 | 2021 | 15.47 |
| 5 | Nakajima et al. | 2017 | Oromia | Health facility | Cross sectional | 642 | 123 | 2013 | 19.16 |
| 6 | Fetene et al. | 2021 | Eastern Ethiopia | Health facility | Cross sectional | 510 | 100 | 2019 | 19.61 |
| 7 | Kedir et al. | 2013 | Eastern Ethiopia | Community | Cross sectional | 1678 | 581 | 2010 | 34.62 |
| 8 | Tesfay et al., | 2018 | Oromia | Health facility | Case control | 336 | 120 | 2013 | 35.71 |
| 9 | Alamneh et al. | 2020 | SNNPR | Community | Cross sectional | 341 | 122 | 2018 | 35.78 |
| 10 | Yadeta et al. | 2020 | Eastern Ethiopia | Health facility | Cross sectional | 1688 | 628 | 2016 | 37.2 |
| 11 | Tesso et al. | 2017 | Oromia | Health facility | Cross sectional | 293 | 193 | 2017 | 65.87 |
