# Supplementary material for: The epidemiology of khat (catha edulis) chewing and alcohol consumption among pregnant women in Ethiopia: A systematic review and meta-analysis
Source: PLOS Glob Public Health. 2023 Sep 15;3(9):e0002248. doi: 10.1371/journal.pgph.0002248 (PMC10503716; doi:10.1371/journal.pgph.0002248)
Supplement: S3 Table — A and B. Study characteristics included in the systematic review and meta-analysis on prevalence of khat and alcohol use among pregnant women in Ethiopia. (ZIP) [file pgph.0002248.s003.zip › S3B_Table.docx]

**S3B Table.** Characteristics of studies included in meta-analysis of alcohol use among pregnant women in Ethiopia.

| **S.No.** | **Primary author and publication year** | **Study period** | **Region** | **Study setting** | **Study design** | **Sample size (qualified rate %)** | **Data collection tool** | **Mean age** | **Case** | **Prevalence** |
| --- | --- | --- | --- | --- | --- | --- | --- | --- | --- | --- |
| 1 | Ahmed et al., 2020 | 2017 | Oromia | Institutional | Cross sectional | 1117 | Dichotomous questionnaire | 25 | 46 | 4.12 |
| 2 | Mekuriaw et al., 2019 | 2017 | SNNPR | Institutional | Cross sectional | 718 | AUDIT | 27.1 | 58 | 8.08 |
| 3 | Fetene et al., 2021 | 2019 | Jigiga, Dire Dawa and Harar | Institutional | Cross sectional | 510 | Dichotomous questionnaire | 26.63 | 48 | 9.41 |
| 4 | Alamneh et al., 2020 | 2018 | SNNPR | Community | Cross sectional | 341 | AUDIT | 28 | 34 | 9.97 |
| 5 | Wubetu et al., 2019 | 2018 | Amhara | Institutional | Cross sectional | 380 | CAGE | 29.6 | 61 | 16.05 |
| 6 | Kassew et al., 2022 | 2016 | National | Community | Cross sectional | 1135 | Dichotomous questionnaire | 22.49 | 255 | 22.47 |
| 7 | Shitie et al., 2023 | 2021 | Amhara | Institutional | Cross sectional | 612 | Dichotomous questionnaire | 28.18 | 161 | 26.31 |
| 8 | Tesso et al., 2017 | 2017 | Oromia | Institutional | Cross sectional | 293 | Dichotomous questionnaire | 26 | 33 | 11.26 |
| 9 | Addila et al., 2021 | 2019 | Amhara | Community | Cross sectional | 1216 | AUDIT | 27.8 | 368 | 30.26 |
| 10 | Anteab et al., 2014 | 2014 | Amhara | Community | Cross sectional | 810 | Dichotomous questionnaire | 26.42 | 275 | 33.95 |
| 11 | Tesfaye et al., 2020 | 2019 | Addis Ababa | Institutional | Cross sectional | 585 | AUDIT | 27.31 | 217 | 37.10 |
| 12 | Bitew et al., 2020 | 2017 | Addis Ababa | Institutional | Cross sectional | 367 | AUDIT | 27.43 | 146 | 39.78 |
| 13 | Abetew et al., 2022 | 2020 | Amhara | Community | Cross sectional | 555 | AUDIT | 25 | 253 | 45.59 |
| 14 | Gelagay et al., 2022 | 2017 | Amhara | Community | Cross sectional | 579 | Dichotomous questionnaire | 31.57 | 336 | 58.03 |
| 15 | Tafese et al., 2022 | 2019 | Amhara | Community | Cross sectional | 228 | Dichotomous questionnaire | 30 | 179 | 78.51 |
| 16 | Demeke et al., 2022 | 2021 | Amhara | Community | Cross sectional | 374 | Dichotomous questionnaire | 35.22 | 287 | 76.74 |
