# Supplementary material for: The epidemiology of khat (catha edulis) chewing and alcohol consumption among pregnant women in Ethiopia: A systematic review and meta-analysis
Source: PLOS Glob Public Health. 2023 Sep 15;3(9):e0002248. doi: 10.1371/journal.pgph.0002248 (PMC10503716; doi:10.1371/journal.pgph.0002248)
Supplement: S4 Table — A and B. Sub-group analysis of khat and alcohol use by different characteristics in Ethiopia. (ZIP) [file pgph.0002248.s004.zip › S4A_Table.docx]

**S4A Table.** Sub-group analysis of studies included in the meta-analysis on the prevalence of khat chewing among pregnant women in Ethiopia.

| Sub-group  variables | Variable category | Included studies | Estimates | I^2^% | *p*-heterogeneity | *p*-difference |
| --- | --- | --- | --- | --- | --- | --- |
|  |  |  | Prevalence (%)95%CI |  |  |  |
| Location | SNNPR | 2 | 22.73 (2.64, 48.10) | 98.8% | *P*<0.001 | *p*<0.001 |
|  | Oromia | 4 | 31.51(9.70, 53.33) | 99.5% | *P*<0.001 |  |
|  | Addis Ababa | 1 | 15.28 (11.56, 19.00) | --- | *---* |  |
|  | Eastern Ethiopia | 4 | 26.75(15.69, 37.80) | 98.7% | *P*<0.001 |  |
| Study setting | Community | 3 | 28.54(14.12, 42.96) | 98.7% | *P*<0.001 | *p*<0.001 |
|  | Institution | 8 | 25.93(14.75, 37.11) | 99.3% | *P*<0.001 |  |
| Sample size | <790 | 7 | 28.61 (16.97, 40.25) | 98.6% | *P*<0.001 | *p*<0.01 |
|  | ≥790 | 4 | 23.26 (7.23, 39.29) | 99.6% | *P*<0.001 |  |
