# Supplementary material for: The epidemiology of khat (catha edulis) chewing and alcohol consumption among pregnant women in Ethiopia: A systematic review and meta-analysis
Source: PLOS Glob Public Health. 2023 Sep 15;3(9):e0002248. doi: 10.1371/journal.pgph.0002248 (PMC10503716; doi:10.1371/journal.pgph.0002248)
Supplement: S4 Table — A and B. Sub-group analysis of khat and alcohol use by different characteristics in Ethiopia. (ZIP) [file pgph.0002248.s004.zip › S4B_Table.docx]

**S4B Table.** Sub-group analysis of studies included in the meta-analysis on the prevalence of alcohol use among pregnant women in Ethiopia.

| Subgroup | | Number of studies | Estimates | | I^2^ | *p*-heterogeneity | *p*-difference |
| --- | --- | --- | --- | --- | --- | --- | --- |
|  |  |  | Prevalence (%) | 95%CI |  |  |  |
| Location | Amhara | 8 | 45.62 | 31.25, 59.99 | 99.2% | *p*<0.001 | *p*<0.001 |
|  | SNNPR | 2 | 8.62 | 6.92, 10.31 | 0% | *p*=0.324 |  |
|  | Oromia | 2 | 7.47 | 0.49, 14.46 | 92.6 | *p*<0.001 |  |
|  | Addis Ababa | 2 | 38.12 | 35.04, 41.21 | 0% | *p*=0.408 |  |
|  | Jijiga, dire dawa and Harar | 1 | 9.41 | 6.88, 11.94 | --- | *----* |  |
|  | National | 1 | 22.47 | 20.04, 24.90 | ---- | ---- |  |
| Study setting | Community | 8 | 44.36 | 29.16, 59.57 | 99.4% | *p*<0.001 | *p*<0.001 |
|  | Health facility | 8 | 18.86 | 10.94, 26.78 | 98.6% | *p*<0.001 |  |
| Data collection tool | AUDIT | 6 | 28.39 | 15.40, 41.38 | 99.0% | *p*<0.001 | *p*<0.001 |
|  | Dichotomous question | 9 | 35.57 | 19.78, 51.35 | 99.7% | *p*<0.001 |  |
|  | CAGE | 1 | 16.05 | 12.37, 19.73 | --- | *---* |  |
| Sample size | <613 | 11 | 37.11 | 22.78, 51.44 | 99.4% | *p*<0.001 | *p*<0.001 |
|  | ≥613 | 5 | 19.73 | 8.06, 31.39 | 99.4% | *p*<0.001 |  |
| Mean age  (year) | <30 | 13 | 22.56 | 15.23, 29.90 | 99.0% | *p*<0.001 | *p*<0.001 |
|  | ≥30 | 3 | 71.03 | 57.61,84.45 | 96.2% | *p*<0.001 |  |
