# Supplementary material for: The epidemiology of khat (catha edulis) chewing and alcohol consumption among pregnant women in Ethiopia: A systematic review and meta-analysis
Source: PLOS Glob Public Health. 2023 Sep 15;3(9):e0002248. doi: 10.1371/journal.pgph.0002248 (PMC10503716; doi:10.1371/journal.pgph.0002248)
Supplement: S5 Table — A and B. Sensitivity analysis showing presence of influential study among studies conducted to determine khat and alcohol prevalence among pregnant women in Ethiopia. (ZIP) [file pgph.0002248.s005.zip › S5A_Table.docx]

S5A Table. Sensitivity analysis of the prevalence of khat chewing among pregnant women in Ethiopia.

| S.No. | Study excluded | Prevalence of khat  chewing | 95% Confidence interval |
| --- | --- | --- | --- |
|  | Ahmed et al., 2021 | 28.73 | 20.18, 37.27 |
|  | Mekuriaw et al., 2020 | 28.34 | 18.53, 38.15 |
|  | Dendir et al., 2017 | 27.78 | 18.22, 37.34 |
|  | Misgana et al., 2022 | 27.78 | 17.75, 37.82 |
|  | Nakajima et al., 2017 | 27.40 | 17.68, 37.12 |
|  | Fetene et al., 2021 | 27.35 | 17.73, 36.97 |
|  | Kedir et al., 2013 | 25.82 | 16.69, 34.96 |
|  | Tesfay et al., 2018 | 25.74 | 16.47, 35.02 |
|  | Alamneh et al., 2020 | 25.74 | 16.47, 35.01 |
|  | Yadeta et al., 2020 | 25.55 | 16.73, 34.38 |
|  | Tesso et al., 2017 | 22.78 | 14.57, 30.99 |
| Combined | | 26.64 | 17.77, 35.50 |
