# Supplementary material for: The epidemiology of khat (catha edulis) chewing and alcohol consumption among pregnant women in Ethiopia: A systematic review and meta-analysis
Source: PLOS Glob Public Health. 2023 Sep 15;3(9):e0002248. doi: 10.1371/journal.pgph.0002248 (PMC10503716; doi:10.1371/journal.pgph.0002248)
Supplement: S5 Table — A and B. Sensitivity analysis showing presence of influential study among studies conducted to determine khat and alcohol prevalence among pregnant women in Ethiopia. (ZIP) [file pgph.0002248.s005.zip › S5B_Table.docx]

S5B Table. Sensitivity analysis of the prevalence of alcohol consumption among pregnant women in Ethiopia.

| S.No. | Study excluded | Prevalence of alcohol consumption | 95% Confidence interval |
| --- | --- | --- | --- |
|  | Ahmed et al., 2020 | 33.49 | 23.47, 43.52 |
|  | Mekuriaw et al., 2019 | 33.23 | 22.52, 43.94 |
|  | Fetene et al., 2021 | 33.14 | 22.60, 43.68 |
|  | Alamneh et al., 2020 | 33.10 | 22.70, 43.50 |
|  | Wubetu et al., 2019 | 32.69 | 22.32, 43.05 |
|  | Kassew et al., 2022 | 32.27 | 21.62, 42.92 |
|  | Shitie et al., 2023 | 32.00 | 21.65, 42.36 |
|  | Tesso et al., 2017 | 33.01 | 22.66, 43.36 |
|  | Addila et al., 2021 | 31.74 | 21.30, 42.19 |
|  | Anteab et al., 2014 | 31.49 | 21.23, 41.76 |
|  | Tesfaye et al., 2020 | 31.28 | 21.12, 41.46 |
|  | Bitew et al., 2020 | 31.11 | 20.98, 41.24 |
|  | Abetew et al., 2022 | 30.72 | 20.71, 40.72 |
|  | Gelagay et al., 2022 | 29.88 | 20.26, 39.50 |
|  | Tafese et al., 2022 | 28.54 | 19.22, 37.86 |
|  | Demeke et al., 2022 | 28.63 | 19.73, 37.53 |
| Combined | | 31.65 | 21.83, 41.46 |
