# Supplementary material for: The epidemiology of khat (catha edulis) chewing and alcohol consumption among pregnant women in Ethiopia: A systematic review and meta-analysis
Source: PLOS Glob Public Health. 2023 Sep 15;3(9):e0002248. doi: 10.1371/journal.pgph.0002248 (PMC10503716; doi:10.1371/journal.pgph.0002248)
Supplement: S6 Table — A and B. Meta-regressions of khat use among pregnant women in Ethiopia by sample size, and publication year of included studies. (ZIP) [file pgph.0002248.s006.zip › S6A_Table.docx]

**S6A Table.** Meta regression of studies included in the meta-analysis on the prevalence of khat chewing among pregnant women in Ethiopia.

| **Category** | **Meta-regression coefficient** | **95%CI** | ***p*-value** |
| --- | --- | --- | --- |
|  |  |  |  |
| **Location** | | | |
| Addis Ababa (ref) | - | - | - |
| Eastern Ethiopia | 0.6472 | -1.7842, 3.0786 | 0.602 |
| SNNP | 0.3166 | -2.3476, 2.9807 | 0.816 |
| Oromia | 0.6747 | -1.7582, 3.1075 | 0.587 |
| **Study setting** | | | |
| Community (ref) | - | - | - |
| Health facility | -0.2765 | -1.5945, 1.0416 | 0.681 |
| **Study period** | -0.1596 | -0.3731, 0.0538 | 0.143 |
| **Sample size** | -0.00022 | -0.0014, 0.0009663 | 0.715 |
