# Supplementary material for: The epidemiology of khat (catha edulis) chewing and alcohol consumption among pregnant women in Ethiopia: A systematic review and meta-analysis
Source: PLOS Glob Public Health. 2023 Sep 15;3(9):e0002248. doi: 10.1371/journal.pgph.0002248 (PMC10503716; doi:10.1371/journal.pgph.0002248)
Supplement: S6 Table — A and B. Meta-regressions of khat use among pregnant women in Ethiopia by sample size, and publication year of included studies. (ZIP) [file pgph.0002248.s006.zip › S6B_Table.docx]

**S6B Table.** Meta-regression of studies included in the meta-analysis on the prevalence of alcohol use among pregnant women in Ethiopia.

| **Category** | **Meta-regression coefficient** | **95%CI** | ***p*-value** |
| --- | --- | --- | --- |
|  |  |  |  |
| **Region** | | | |
| Addis Ababa (ref) | - | - | - |
| Amhara | 0.2746 | -1.1441, 1.69323 | 0.704 |
| SNNP | -1.845576 | -3.6483, -0.0428 | 0.045* |
| Oromia | -2.13806 | -3.9422, -0.3338 | 0.020* |
| Eastern Ethiopia | -1.792856 | -4.0020, 0.4162 | 0.112 |
| National | -0.7671502 | -2.9606, 1.4263 | 0.493 |
| **Study setting** | | | |
| Health facility (ref) | - | - | - |
| Community | 1.3997 | 0.3348, 2.4645 | 0.010* |
| **Study period** | 0.2275 | -0.1142, 0.5691 | 0.192 |
| **Sample size** | -0.0013 | -0.0033, 0.0007 | 0.208 |
| **Data collection tool** | | | |
| AUDIT (ref) | - | - | - |
| Dichotomous question | -0.5603 | -1.1422 1.6462 | 0.701 |
| CAGE | 0.2520 | -3.4208 2.3002 | 0.723 |
| **Mean age (year)** | .25795 | 0.0747, 0.4412 | *P<0.01** |
